# Transparent Figures: Researching and Preserving Objects of Cellulose Acetate

**DOI:** 10.3390/polym15132838

**Published:** 2023-06-27

**Authors:** Benjamin Kemper, Christoph Herm

**Affiliations:** Study Program of Restoration, Dresden University of Fine Arts, 01307 Dresden, Germany

**Keywords:** Deutsches Hygiene-Museum, cellulose acetate, additive, degradation, preservation, museum climate, relative humidity, color change, reaction kinetics

## Abstract

In 1935, the Deutsches Hygiene-Museum Dresden began to produce the so-called Transparent Figures, which became icons of the 20th century. This study aims to explore the effects of external agents such as humidity and temperature on the aging mechanism of the materials of the Transparent Figures and to slow it down through preventive measures. The focus is on cellulose acetate (CA), which was used for the outer skin of the Transparent Figures. The original objects were investigated using FTIR, Raman, and GC–MS. On some Transparent Figures, liquid leakage of additives occurs when the relative humidity rises above 50–60% RH and is accompanied by a release of acetic acid. Based on these findings, original CA used for the production of the Transparent Figures was artificially re-aged at 70 °C while varying the relative humidity. The specimens were analyzed with colorimetry and GC–MS. Additive content, degree of substitution and degree of polymerization were determined. The results showed that the degradation is slowed down at 30% RH compared to aging at 50% RH or 70% RH. Thus, lowering the relative humidity seems effective in slowing down the degradation of the CA of the Transparent Figures. A relative humidity of 30% RH and a temperature of 15 °C are recommended.

## 1. Introduction

The studies presented here were prepared as part of a project to research the long-term preservation of the so-called Transparent Figures of the Deutsches Hygiene-Museum Dresden (DHMD) [1]. They are the first life-size teaching models of the human and some animal bodies and were produced at the DHMD for many decades from 1930 onwards [2]. Their special feature is the transparent outer skin, which allows an extensive view of the inside of the body. The outer skin is made of the then novel polymer cellulose acetate (CA), which is made processable by additives. As is typical for CA, the Transparent Figures show signs of aging in the form of embrittlement, shrinkage and yellowing.

In the following, the general degradation of CA is reviewed, as it can also be observed on the Transparent Figures. During the degradation of CA, acetic acid is released, which in turn induces degradation reactions on other materials in the near environment and acts on the degradation of the CA itself. Furthermore, additives migrate from the CA and can also induce further damage [3]. Despite this knowledge, the exact reaction mechanisms of the degradation of CA and their dependencies on external agents such as humidity and temperature have not been fully explored. Much research on this has been conducted using animation slides and photographic films [4,5,6,7,8,9]. However, the Transparent Figures are large three-dimensional objects made of CA with a thickness of up to 2 mm, and therefore are distinguished from objects made from thinner foils and films. This present study therefore aims to investigate the degradation of the CA of the Transparent Figures and to develop preventive measures to slow it down as much as possible. For this purpose, it was possible to observe the aging behavior of the CA from different decades, to take samples from selected original figures and to perform specific measurements in and around the objects. These findings are used to perform artificial aging at elevated temperature as a function of external agents such as relative humidity (RH) or acetic acid concentration. The goal is to predict the natural aging behavior [10]. Due to the access to residual materials from the workshops of the DHMD, original, naturally aged CA was used for the model experiments, so it can be assumed that the results of the analyses and experiments are close to the phenomena of the Transparent Figures. Subsequently, the chemical composition and color of the artificially re-aged CA are analyzed and the effects of the external agents on these are determined. Finally, investigation results on the Transparent Figures are compared with the findings from the model tests. Theoretical considerations of the effects of the external agents on the service life of the CA complement this research. All findings are incorporated into a concept for preventive conservation, which contains suggestions for slowing down the degradation of the CA of the Transparent Figures.

CA gained great importance as a carrier for film materials [11,12] and for the production of animation films [12]. CA is also found in other products such as textile fibers [13], eyeglass frames, combs or even toys from the beginning of the 20th century [14,15,16]. The wide distribution, good processability, as well as its transparency were among the reasons why CA was used for the Transparent Figures. For the production of CA raw stock, first the cellulose triacetate is synthesized; if desired, the cellulose diacetate is then synthesized by partial hydrolysis [17,18]. Even though the latter could be processed better, both variants became attractive for application only after the addition of additives. The properties of the CA, such as solubility and thermal behavior, essentially depend on three factors, which lead to a great diversity of the material [19]:

- degree of substitution (DS), corresponding to the acetyl content
- degree of polymerization (DP), corresponding to the chain length
- type and content of additives

The choice of additives depends strongly on the intended use of the CA. Common additives are phthalates, esters of aliphatic dicarboxylic acids, phosphoric acid esters, alkyl sulfonic acid esters or polyesters, especially dimethyl phthalate (DMP), diethyl phthalate (DEP) and triphenyl phosphate (TPP) [20]. They are widely used, for example, in films, injection molding compounds, and even biodegradable products [21]. The low-cost phthalates serve as plasticizers and TPP as a flame-retardant plasticizer [19]. Furthermore, triacetin (glycerol triacetate) is frequently used, e.g., in the classic cigarette filter [21].

Five main processes are responsible for the degradation of CA: hydrolysis, chain scission, additive loss, oxidation and photodegradation [17]. Hydrolysis of the acetyl groups (deacetylation) is the most characteristic step. In combination with water from the air, acetic acid is formed, which is perceived as a strong vinegar odor. Therefore, this phenomenon is also called “vinegar syndrome”, which often appears on CA objects before any visual change [22,23]. Hydrolysis decreases the degree of substitution of CA. Deacetylation reactivity at the three acetylated positions decreases in the order of C2, C6, C3, which is probably related to interactions with neighboring groups [24,25]. Various research papers show that increased relative humidity has an influence on CA deacetylation [26,27] and therefore is treated experimentally in the present work. The released acetic acid has further negative effects on the CA itself. It catalyzes chiefly deacetylation so that the DS is reduced. As a result, increased moisture absorption is possible due to the higher amount of hydroxy groups [17]. Furthermore, a volume reduction of the CA is caused by the outgassing of acetic acid. Due to the loss of acetyl groups, the physically bound additives increasingly migrate out of the CA [7]. The loss of the additives also causes shrinkage of the CA and brittleness. The surface becomes sticky with the remaining additives [17]. Additives can also themselves contribute to accelerating degradation through reaction products [28]. Furthermore, the often used TPP recrystallizes in CA after loss of acetic acid and co-plasticizer, sometimes leading to shattering of the polymer [29]. Oxidation and photodegradation are caused by oxygen and light. Oxidation releases smaller molecular fragments, such as formic acid or oxalic acid [17]. In photodegradation, the main products are carbon monoxide, carbon dioxide and methane, primarily formed by the radical cleavage of esters [30,31]. Oxidations, photodegradation as well as thermal influences contribute to the yellowing of the CA. Presumably, this is favored by the formation of carboxyl and carbonyl groups [32], while the decomposition of the additives could also contribute to this [31].

For the preservation of CA, a low temperature and a low relative humidity are generally recommended. As there are no generally valid numerical values for this, many and very different approaches are followed. For example, Allen recommends a dry atmosphere and temperatures below 0 °C [26]. In March 2006, the Danish National Cultural Heritage Agency recommended a temperature of 2–5 °C and a relative humidity of 20–30% [28]. For film materials, the Image Permanence Institute (IPI) recommends a temperature between 0 and 20 °C and a relative humidity of 30–50% [33]. Nevertheless, there is a great need for research on recommendations in the handling of CA because, as mentioned, CA is used for many different applications in a wide variety of forms, thicknesses and combinations with other materials.

Artificial aging attempts to accelerate the course of naturally occurring decomposition processes of materials, whereby the aging process can be better estimated and monitored. Suitable measures derived from this can lead to a significant extension of the service life of the material [10]. For the aging of CA, temperature and relative humidity are particularly considered in this work. The influence of temperature on the reaction rate constant of chemical reactions can in many cases be described at least approximately by the well-known equation of Svante Arrhenius [34]. This model was used especially by Adelstein et al. [4,5,6,8] to explore the effects of relative humidity on a wide variety of materials. Later, this method was standardized in ISO 18924 [35] and is now a useful way to develop preservation strategies for film and paint materials [27]. However, the Arrhenius approach also has disadvantages. It assumes that the chemical reactions and physical processes occur at the use temperature in exactly the same way as at the elevated temperature used for accelerated aging. Similarly, the activation energy is assumed to be independent of temperature. Therefore, as soon as the reactions and processes become more complex, the Arrhenius equation reaches its limit [36]. Therefore, other models exist that complement the thermal approach, such as the so-called isoconversional approach [37]. In conservation science, the model of Michalski has been introduced [38,39]. With this, starting from the Arrhenius equation, calculations can be carried out for the reaction rate or for the service life, taking into account different levels of relative humidity and different activation energies for hydrolytic reactions.

## 2. Materials and Methods

### 2.1. Material

#### 2.1.1. Historical Transparent Figures

The Transparent Figures are probably the best known objects from the workshops of the DHMD. The first Transparent Men were presented to the public as early as 1930 [2]. Over seven decades, a total of more than 130 Transparent Figures were produced. The first Transparent Men were soon followed by Transparent Women, Transparent Cows and Transparent Horses [40]. The Transparent Figures are life-size and have been exhibited in various contexts around the globe. In most cases, they served as teaching aids to illustrate the structure of the replicated bodies. Their transparent outer skin is usually made of CA, allowing a view of the skeleton, organs, nerves and blood vessels. Furthermore, the CA was replaced by cellulose acetate butyrate CAB in the 1980s as material for the production of the Transparent Figures [41]. A wide variety of materials were used to create the inner body. For example, the skeleton is made of aluminum or hydronalium. Veins, arteries and nerves were made of twisted copper wire, which was then tinned and coated with colored alkyd resins [41,42,43]. For a few blood vessels and nerves, lead was used instead of copper wire. The organs were made of CA and polymethyl methacrylate (PMMA) and coated with nitrocellulose dip varnish. In addition, steel screws, telephone wires, incandescent lamps, rubber, and PVC were used for the skull and kneecap [41,42]. The result is a complex mix of diverse materials, the combination of which was a technical and artistic masterstroke. In addition, the organs could be illuminated individually, and an audio lecture complemented the presentation of the objects. In the following section, the four Transparent Figures relevant to this work are briefly introduced. Observations on these objects, as well as the possibility of taking samples and determining degradation products, provided valuable insights into the degradation of CA.

**Figure 1:**
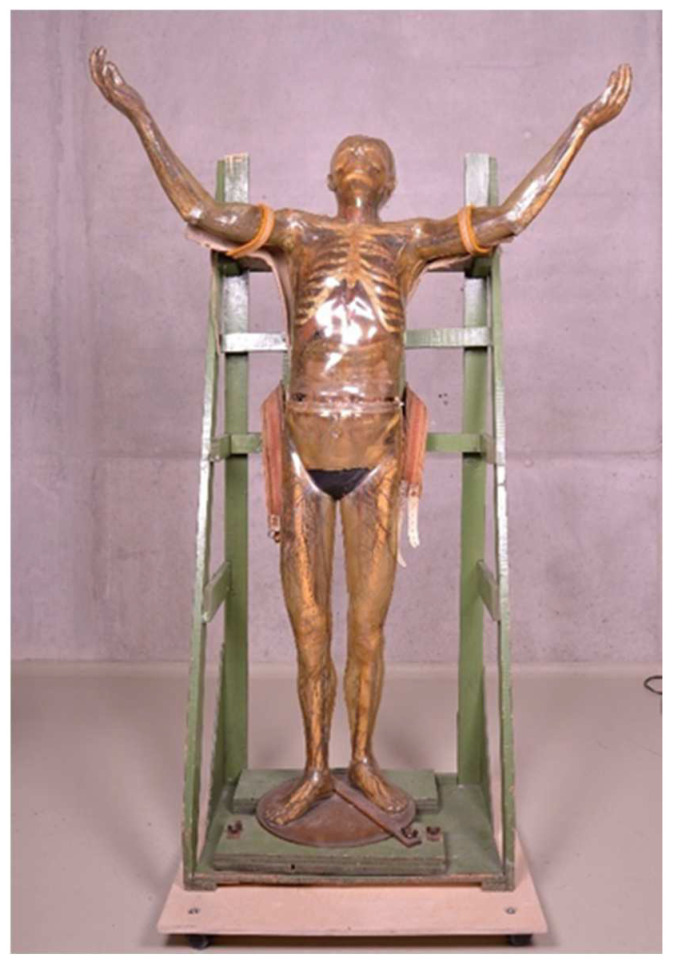
Transparent Man from 1935 in his original wooden transport rack.

The Transparent Man from 1935 (inventory number: DHMD 2011/38) (Figure 1) is the fifth Transparent Figure ever made in the DHMD workshops. It was presented in the exhibition “Mother and Child” in Stockholm, which opened on 10 January 1936. Subsequently, the object was shown in several exhibitions and toured Europe for many years as part of a showman’s enterprise. Since 2009 the object has again been in the possession of the DHMD [44]. The outer skin of CA is greenish discolored, heavily shrunken and brittle. It is also cracked in several places. The figure shows heavy soiling [45]. The Transparent Man from 1935 can be split at the waist into upper and lower halves.

**Figure 2:**
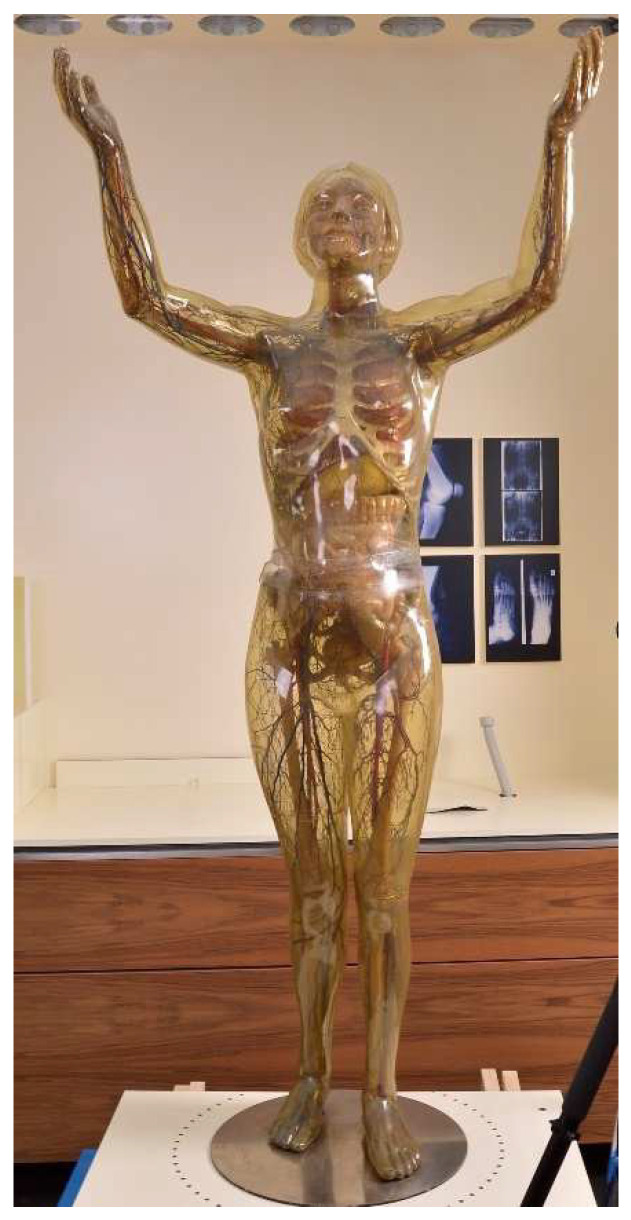
Transparent Woman from 1935 in the DHMD in the year 2018.

**Figure 3:**
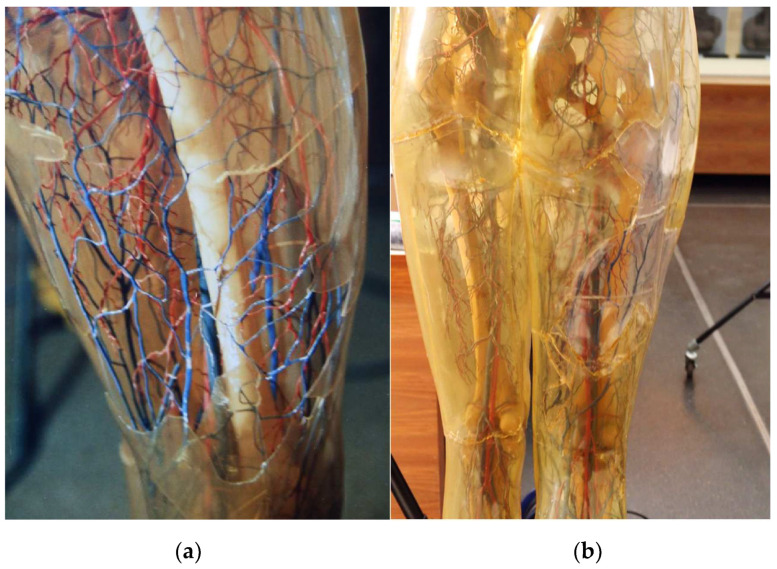
Transparent Woman from 1935, details: back upper leg, (**a**) before restoration in 1990. A big part of the outer skin is missing. (**b**) After restoration in 2018. The difference between supplemented (transparent) and original (yellowed) CA is clearly visible. Such additions were also made to the head and hip of the figure.

The Transparent Woman from 1935 (inventory number: DHMD 2002/1L) (Figure 2) is the first Transparent Woman produced and was presented at the Museum of Science in New York in 1936. She subsequently toured the United States for many years and came to the Deutsches Historisches Museum Berlin in 1988 from her previous location at the Science Museum in St. Louis [44]. In 1990, she was extensively restored at the DHMD. Among other things, various parts of the skin were refixed or replaced [41,42,46]. These measures can still be seen today (Figure 3a,b). As a permanent loan, it has been on display in the permanent exhibition of the DHMD since 2004. The outer skin of the Transparent Woman is almost completely closed. There is only a very narrow gap at the hip and a small crack on the left foot. On the right side at the level of the waist, there are visible liquefactions on the intestine [47]. The Transparent Woman 1935 can be divided into upper and lower halves at the waist. From the Transparent Woman from 1935 no samples were taken because it is not owned by the DHMD.

**Figure 4:**
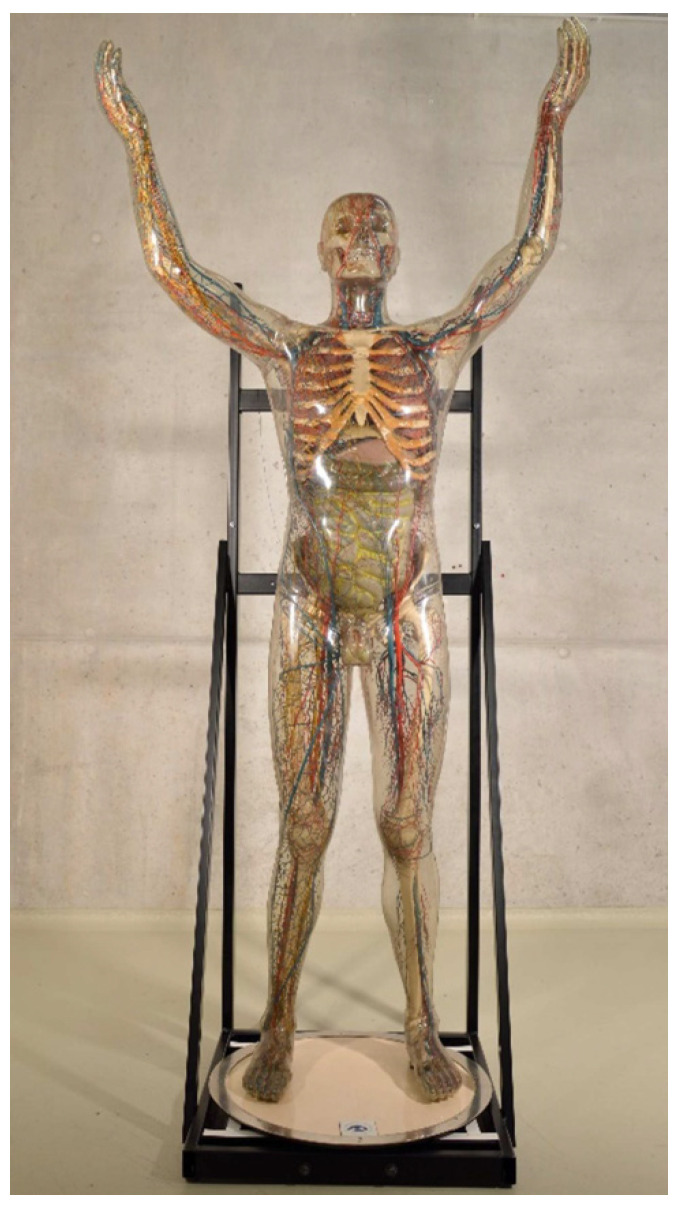
Transparent Man from 1962.

The Transparent Man from 1962 (inventory number: DHMD 1994/520.1) (Figure 4) was sold to the Paul Stradins Museum of Medical History in Riga in 1963. The figure was exhibited there until the early 1990s before it was returned to the DHMD in exchange for another Transparent Figure. Since then he has been shown in various special exhibitions in Germany and Europe [48]. The CA outer skin of the transparent Man from 1962 is clear and shows no yellowing. It is completely closed. Only in the neck area are there two small revision flaps for unscrewing. Except for a slight corrosion on screws of the sternum, there are hardly any noteworthy conspicuous features. Unlike the previously described figures, the Transparent Man from 1962 is not separable at the waist.

**Figure 5:**
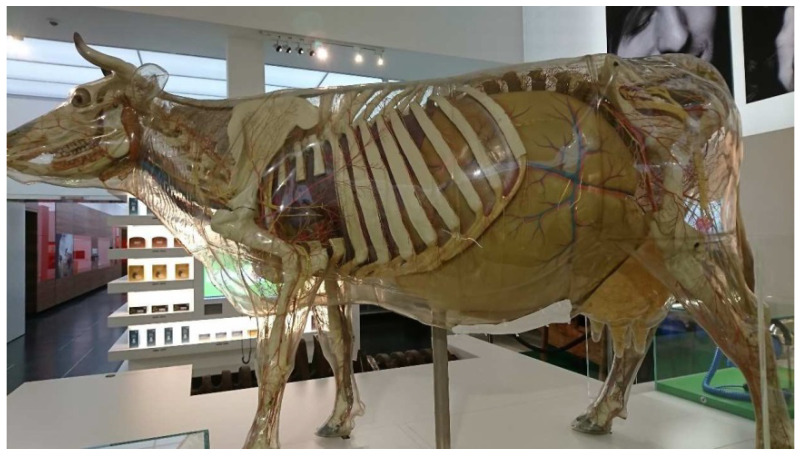
Transparent Cow from 1983 in the permanent exhibition of the DHMD.

**Figure 6:**
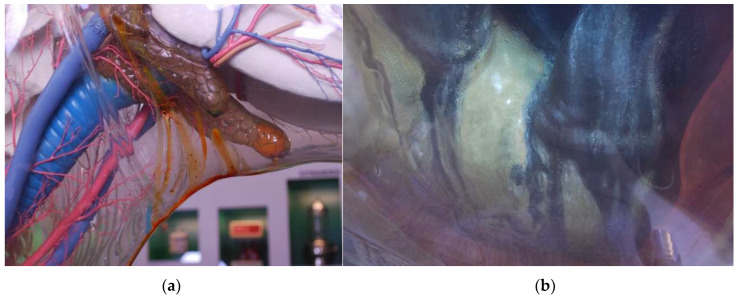
Transparent Cow from 1983, details. (**a**) Exudated liquid under the mandible. (**b**) Droplet formation on the fetus.

The Transparent Cow from 1983 (inventory number: DHMD 2001/89) (Figure 5) is the last one of a total of nine Transparent Cows that have been produced at the DHMD since 1956. It remained in the DHMD and has been exhibited in several special exhibitions in Germany and Austria. Since 2004/2005 it has been part of the permanent exhibition of the DHMD [49]. The Transparent Cow from 1983 has a transparent outer skin made of CA without significant yellowing. It is almost completely closed. It has two smaller inspection flaps and a large one on the underside. The interior shows strong degradation phenomena, which are important for conclusions in the further course of this work: Fluid migrates from the parotid and mandibular glands and runs down the inside of the outer skin (Figure 6a). The progression of this process can be observed within a short time span of a few weeks and months. Furthermore, liquid drops form on the fetus in the rear right area of the Transparent Cow shown as pregnant (Figure 6b). This drop formation occurs in the course of a few months, sometimes strongly, sometimes less strongly. Temporally, an almost complete drying of the drops can also be observed. According to the DHMD staff, these phenomena occurred for the first time in 2011/2012 and have continuously worsened since then [50].

#### 2.1.2. Cellulose Acetate

**Figure 7:**
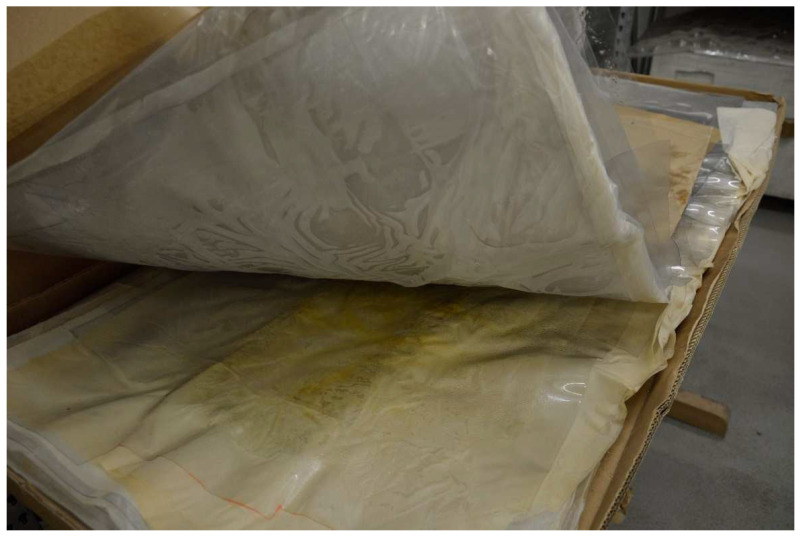
Sheet material made from CA and cellulose acetate butyrate, stored in cardboard boxes in the workshop of DHMD.

For the aging studies, original transparent sheet material was used, which dates from around 1980 and probably was used to create the cow. The earlier CA materials of the three human bodies have a different behavior, as can be observed in the different photographs (Figure 2, Figure 3, Figure 4 and Figure 5) and have different additive content as compared to the sheet material as well as to each other (see results of analysis below). The sheets have been stored in cardboard boxes in the DHMD workshop since the 1980s (Figure 7). The climatic conditions have not been recorded, but normal indoor environmental conditions can be assumed. It is unknown whether and how often the cardboard boxes were opened. The plates are colorless and transparent. Some of them are warped and there is a strong odor of acetic acid emanating from them. Furthermore, a liquid has migrated from the sheet material. They can therefore be described as clearly aged. Stacking of the sheets did not prevent plasticizer from escaping from the material. However, the general state of degradation seemed not worse than that of the Transparent Figures.

**Figure 8:**
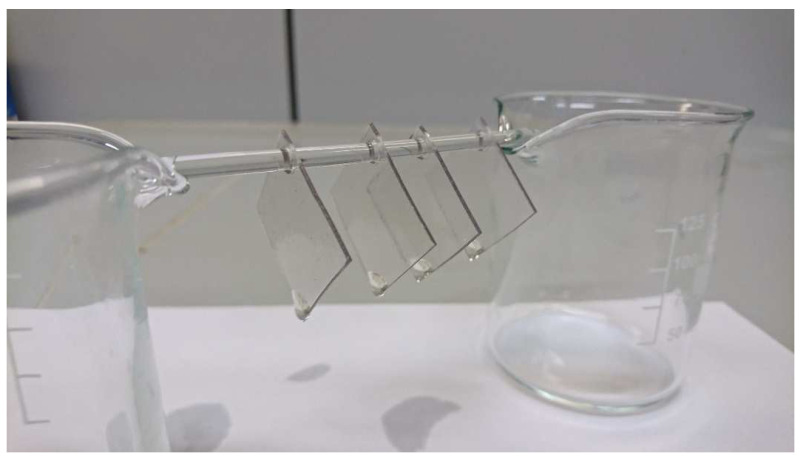
CA test specimens after exposure to air containing 35 ppm acetic acid after five weeks.

It is important to be able to distinguish between CA and CAB because these two materials are stored together. The CA has to be selected as the starting material for the artificial aging experiments in this study, because all investigated Transparent Figures are made of this polymer, as shown by analyses, except for the Transparent Woman from 1935. For the following investigations, specimens measuring 2.5 cm × 2.5 cm were made from one of the large, coherent CA plates after identification. A hole at one corner is used for threading onto a glass rod so that the specimens can be aged in a hanging position (Figure 8). The surface of each specimen is cleaned with an ethanol-soaked precision tissue. To validate the determination of the DS and as a comparative substance for the DP, a cellulose triacetate (CTA) standard from Acros Organics without additives was used. For validation only of the determination of the DS, cigarette filters made of CA from the company Gizeh, which contain the plasticizer triacetin, are also used. Besides the mentioned materials, CA is taken from the outer skin of selected Transparent Figures (Table 1). The sample mass is limited to a few mg in order to avoid optical damage to the Transparent Figures. Table 2 gives an overview of the materials and the tests carried out on them.

### 2.2. Characterization of CA and CAB

Since original material of Transparent Figures is also analyzed, a clear distinction is important. For a clear distinction of CA and CAB, Fourier transform infrared spectroscopy (FTIR) and Raman spectroscopy are applied to thee sheet material after extraction of the additives. Material samples of the Transparent Figures are also analyzed under the same conditions.

Fourier-transform infrared spectroscopy (FTIR): A Bruker Tensor-27 FTIR spectrometer coupled to a Hyperion 2000 microscope was used. Samples were analyzed in transmission in a diamond cell. Spectra were acquired in the range 4000–580 cm^−1^ with a resolution of 2 cm^−1^ and 32 scans per spectrum.

Raman spectroscopy was performed directly on material specimens using a dispersive Horiba Jobin Yvon XPlora Raman microscope equipped with 10×, 50× and 100× objectives and a charge-coupled device (CCD) detector. A diode-pumped solid-state laser and a continuous-wave diode laser, emitting light at 532 nm and 785 nm, respectively, were used as excitation sources. A holographic grating of 1200 rulings/mm provided a dispersion of 14.2 cm^−1^ (532 nm) or 6.5 cm^−1^ (785 nm). Output laser powers were p ≤ 50 mW for 532 nm and p ≤ 12 mW for 785 nm, adjusted to the Raman response of the different samples.

### 2.3. Characterization of Liquids Emerging from CA

Water is detected with self-prepared, dehydrated CoCl_2_-paper. The pH value is determined with indicator sticks (Merck Acilit 109531, Merck KGaA, Darmstadt, Germany) after adding a small drop of distilled water. The emerging liquid is collected in a 300 µL vial with screw cap. Approximately 10 µL is diluted with 1 mL methanol and analyzed by gas chromatography–mass spectrometry (GC–MS) for additives and DS.

### 2.4. Additive Content

The quantitative determination of the additives is carried out by GC–MS. For this purpose, the additives must be completely extracted from the CA. A dedicated ultrasonic extraction method developed in this project was applied [51]. On a fresh fracture edge of the CA sample, small chips are scraped off with a scalpel and dried at 60 °C for 3 h. Of these, 20–25 mg are weighed into a dried 12 mL vial with an accuracy of ±0.1 mg and extracted with 5 mL methanol. The extract made up to 25 mL is analyzed. For each aging step as well as for the references, four CA samples are extracted, twice each. The mean value is calculated from the eight results, corresponding to each data point in the following figures. GC–MS parameter: Instrument: Shimadzu QP2020 (Shimadzu, Kyoto, Japan) with auto injector AOC-20i. Injected volume 1 µL; purge solvent methanol. Column: Shimadzu SH RTX 5 ms (DB5), 30 m × 0.25 mm, ds = 0.25 µm. Carrier gas He flow 1.2 mL/min, split 1:10. Temperature program: 80 °C/1 min, 12 K/min up to 140 °C, 8 K/min up to 270 °C, 40 K/min up to 320 °C, 320 °C/7 min. Software Lab Solutions GCMS solution V4.45, Shimadzu Corporation.

The additive content is calculated for all additives as a concentration-dependent variable with the non-specific unit UA (unit area) per mg of initial weight. In addition, the two additives, DMP and TPP, are quantified by an external calibration and their contents are given as percentages. These are chosen because DMP has the aforementioned strong migration from the CA and TPP has a high content in the material. For this purpose, calibration curves are generated from five concentrations between 50 and 250 mg/L intersecting the zero point (Figure S1). The coefficients of determination of the straight lines were R^2^ = 0.9927 for DMP and R^2^ = 0.9979 for TPP.

### 2.5. Degree of Substitution (DS)

The DS is determined by GC–MS preceded by derivatization of CA, forming acetylpyrrolidine (ACP), following [9,12]. CA chips previously extracted and dried at 60 °C are used for derivatization. Of these, 1.5–2.5 ± 0.1 mg are derivatized with 1 mL of pyrrolidine containing 500 ppm of N-methyl-2-pyrrolidone (NMP) as an internal standard. The mixture is heated in a sealed 1.5 mL vial at 80 °C for 24 h in a sand bath. The determination is carried out by GC–MS: Instrument: Shimadzu QP2020 with auto injector AOC-20i. Injected volume 1 µL; purge solvent isooctane. Column: Shimadzu SH RTX 5 ms (DB5), 30 m × 0.25 mm, ds = 0.25 µm. Carrier gas He flow 1.2 mL/minute, split 1:50. Temperature program: 80 °C/1 min, 12 K/min up to 140 °C, 8 K/min up to 270 °C, 40 K/min up to 320 °C, 320 °C/7 min. Software Lab Solutions GCMS solution V4.45, Shimadzu Corporation. Solutions of ACP at six concentrations between 125 and 3000 ppm, each with 500 ppm of NMP as internal standard, are used for calibration. The calibration line is calculated using the ratio of the peak areas of ACP and NMP against the concentration ratio of ACP and NMP (Figure S2). The coefficient of determination of the straight lines was R^2^ = 0.9968 with a slope of y = 1.01. The content of ACP is then calculated via the calibration function. The mass fraction of acetyl in % is calculated according to Equation (1):

(1) $$w_{acetyl}\left(\%\right)=\frac{38.02\%\cdot \rho (acetyl\;pyrrolidin\;\left(ppm\right))\cdot V(pyrrolidin(mL))}{m(CA\left(µg\right))}$$

with 38.02% = acetyl content in ACP.

The DS is calculated according to Equation (2).

(2) $$DS=\frac{3.86\cdot w(acetyl\left(\%\right))}{102.4\%-w(acetyl\left(\%\right))}$$

Four derivatizations are performed for each aging stage and the mean values are calculated. The measured absolute values of DS of the naturally aged CA specimens are very low, at about 0.4. In order to check whether this is a systematic error of the method despite low standard deviations, CA with a known degree of substitution is derivatized in the same way. Four derivatizations of each material are performed and the arithmetic mean is calculated. The results of the analysis agree with the expected values (Table 3). Thus, there is no systematic error in the method.

### 2.6. Degree of Polymerization (DP)

The DP was determined following [52]. First, the weight-average molecular masses Mw and number-average molecular masses Mn are determined by gel permeation chromatography (GPC). For this purpose, the extracted CA samples are dissolved in dimethylacetamide containing 9% LiCl at 90 °C for about 12 h. The volume of the extraction solution was chosen to give a sample concentration of 3 mg/mL for the respective sample mass; 20 µL of the solution is used for the determination. A duplicate determination is performed. GPC set-up: HPLC pump 1200 from Agilent Technologies 1200 (Agilent, Santa Clara, CA, USA), separation column PL PolarGel-M (300 mm × 7.5 mm) from Agilent Technologies, injection volume 20 µL, solvent DMAc + 3 g/L LiCl, flow 1 mL/min, refractive index detector K-2301 from Knauer (Berlin, Germany). Polystyrene standards with Mw of 377,400 u, 96,000 u, 20,650 u, 5460 u and 1300 u are used for calibration. The DP is calculated from Mn and the DS according to Equation (3):

(3) $$DP=\frac{M_{n}}{\left[159+43\times DS+\left(3-DS\right)\right]}$$

### 2.7. Colorimetry

The color difference and the brightness difference in the CIEL*a*b* color space are determined in comparison to a non-aged reference according to ISO 11664-4 [53]. A KonicaMinolta CM-2600d colorimeter with a measuring aperture of 3 mm is used (KonicaMinolta Sensing, Nieuwegein, The Netherlands). The instrument is calibrated with the standards for white and black supplied by the manufacturer. The measurements are made according to the SCI method (Size Component Included). A white sheet of paper is used as the background for the transparent samples. The colorimeter measures three times in succession and then displays the average value. Each reference and each artificially re-aged CA sample are measured twice. The color differences ∆E*a,b and the brightness differences ∆L* are calculated [53] and related to the average of eight measurements on four references. A color measurement could not be performed for every artificially re-aged CA specimen, since some of them were heavily crumbled.

### 2.8. Acetic Acid in the Air

The acetic acid concentration in air is determined using Dräger diffusion tubes Acetic Acid 10/a-D (Dräger, Lübeck, Germany, #8101071). In the presence of acetic acid, a color change from blue-violet to yellow occurs. With this passive sampler system, concentrations of 1.3–200 ppm can be determined, depending on the measurement duration (1–8 h).

### 2.9. Artificial Aging of CA

#### 2.9.1. Variation of Relative Humidity at Elevated Temperature

Despite its disadvantages and for reasons already mentioned in the introduction, the purely thermal model according to Arrhenius is initially used in this work to carry out accelerated aging. In the present work, the temperature is increased and the relative humidity is varied at the same time. This allows the influence of relative humidity on the CA to be considered separately. For this purpose, the temperature of artificial aging is based on the experimental setup of Richardson et al. [7], who specified 70 °C for aging CA from animation films. The formula of Michalski [38,39] (Equation (4)) is used as the basis for calculating the “lifetime”. For this purpose, the activation energy is required. For the hydrolysis reaction of CA, this is 90.3 kJ-mol^−1^ [7].

(4) $$L_{rel}={{\left(\frac{50\%}{RH}\right)}^{1.3}e}^{\left[\frac{E_{a}}{R}\left(\frac{1}{T_{2}}-\frac{1}{293}\right)\right]}$$

with:

L_rel_: relative lifetime (related to 20 °C and 50% RH)

RH: final relative humidity (%)

T_2_: final temperature (K)

Ea: activation energy = 90.3 J·mol^−1^)

R: universal gas constant = 8.314 J·K^−1^·mol^−1^

This allows the relative lifetime to be calculated based on 20 °C and 50% RH. The reciprocal of the relative lifetime is the factor of the reaction rate relative to 20 °C and 50% RH (Table 4). Table 5 gives an overview of the artificial aging time in years according to the Michalski model, simulated in this study and calculated with the values from Table 4.

The CA specimens suspended from a glass rod (Figure 8) are artificially re-aged in a climatic test chamber at a constant temperature of 70 ± 0.5 °C and under variation of the relative humidity of 30, 50, and 70 ± 1–4% RH for a period of 16–82 days. Device: KPK 120, mytron Bio- und Solartechnik GmbH.

#### 2.9.2. Variation of Acetic Acid Content in the Air

Artificial aging of suspended CA specimens under variation of acetic acid concentrations in air is performed in three desiccators with volumes of 0.6–4.35 L. Four CA specimens are used for each concentration of acetic acid as well as for the reference. Depending on the concentration to be adjusted and the volume of the desiccator, a suitable amount of acetic acid is added to the desiccator and the lid is closed. Due to evaporation, the calculated concentrations of 0.3/1.0/2.0/10/25/35/50/70 ppm are set. To ensure a constant concentration of acetic acid in the air and relative humidity of 40–50% RH, the desiccators are opened every 2–3 days, briefly ventilated and then acetic acid is added again. The temperature is between 22–24 °C during this time. The duration of artificial aging is five weeks.

## 3. Results

### 3.1. Characterization of CA and CAB

**Figure 9:**
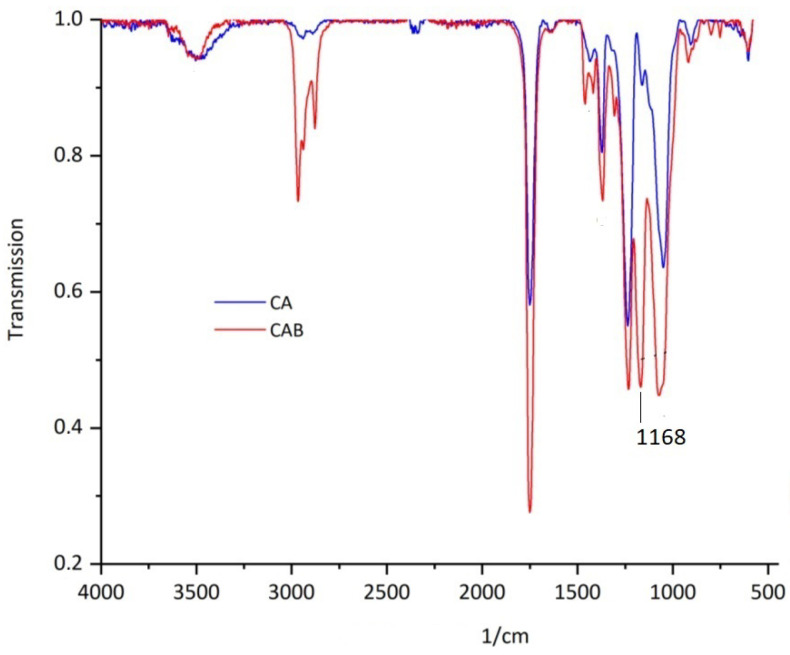
FTIR spectra of extracted CA and extracted CAB.

**Figure 10:**
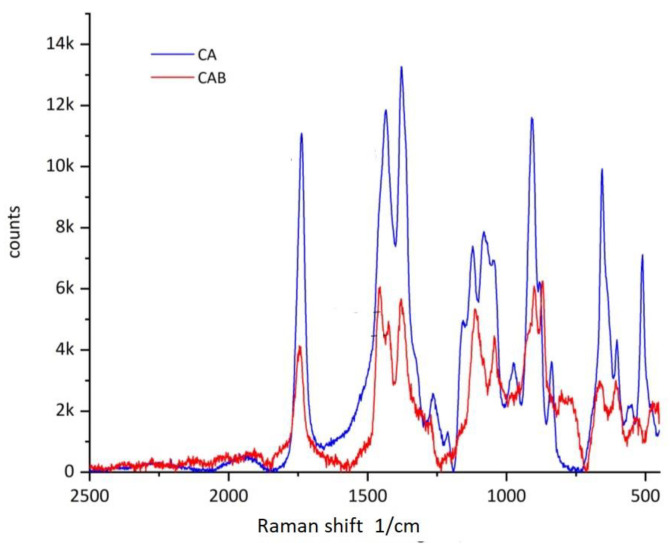
Raman spectra of extracted CA and extracted CAB.

FTIR and Raman spectra of extracted CA and CAB are recorded and contrasted to differentiate the historical sheet material. These materials are readily distinguishable from each other in a combination of FTIR and Raman spectroscopy. In the FTIR spectrum (Figure 9), discrimination is possible by the intensity of the C-O-C valence vibration at 1168 cm^−1^ [54]. In the Raman spectrum (Figure 10), discrimination is possible at a Raman shift between 1377–1454 cm^−1^ and in the range 2800–3000 cm^−1^ [55]. By using these two spectroscopic methods, it is possible to clearly distinguish the plate material from the DHMD workshops as well as to characterize the original material from the Transparent Figures. It is shown that the outer skin of all the Transparent Figures mentioned in this paper is exclusively CA [42]. The above-mentioned Transparent Cow from 1983 could therefore be one of the last Transparent Figures for which CA was used.

### 3.2. Naturally Aged CA

#### 3.2.1. Analysis of Transparent Figures

**Figure 11:**
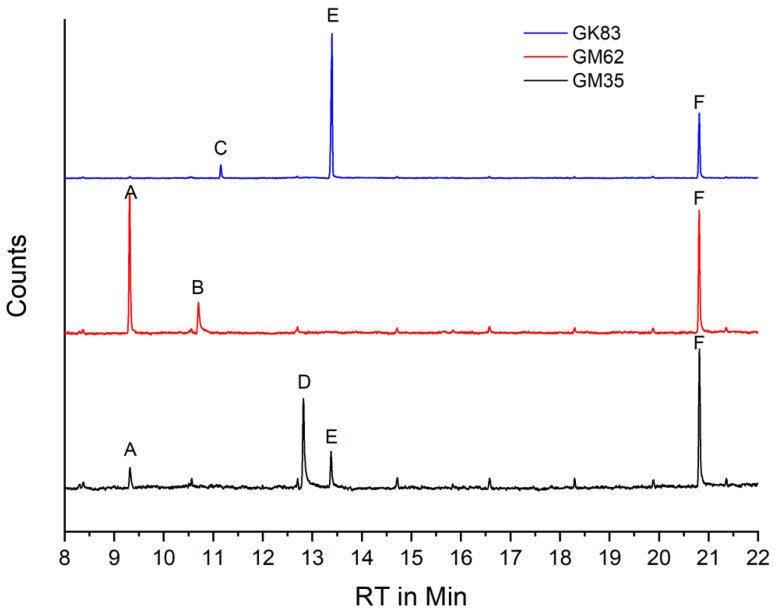
Total ion chromatogram (TIC) of the methanol extract of CA sampled from the Transparent Man from 1935 (GM35), Transparent Man from 1962 (GM62), and Transparent Cow from 1983 (GK83). Additives: A= dimethylphthalate (DMP), B = N-methylbenzensulfonamide (MBSA), C = diethylphthalate (DEP), D = N-ethyl-4-methylbenzensulfonamid (EMSA), E = tris(2-chloroethyl)phosphate (TCEP), F = triphenylphosphate (TPP).

From three of the four Transparent Figures (Table 1), the original CA is analyzed qualitatively by GC–MS. The sample mass is not sufficient for quantification of the additives. The CA of the Transparent Figures contains three to four additives each. The CA of the Transparent Figures have different additive compositions (Figure 11). Only triphenylphosphate (TPP) is contained in all figures. The Transparent Man from 1935 contains N-ethyl-4-methylbenzensulfonamid (EMSA), a small amount of dimethylphthalate (DMP), and tris(2-chloroethyl)phosphate (TCEP). The Transparent Man from 1983 contains DMP and a small amount of N-methylbenzenesulfonamide (MBSA). The Transparent Cow from 1983 contains mostly TCEP and little diethylphthalate (DEP). None of the three figures examined corresponds to the CA specimens (see below). There are three additives in common between the Transparent Figures and the CA specimens, but in different combinations (Table 1). This means that the material cannot come from the same manufacturing process. The sources from which the respective CA for the production of the Transparent Figures was obtained are still the subject of further research.

The degree of substitution (DS) was then determined from the extracted original CA (Table 6). The Transparent Man from 1935 shows a lower DS than the Transparent Cow from 1983 and the Transparent Man from 1962. The absolute values are significantly higher than could be assumed. For example, the DS of the brittle, shrunken, and yellowed CA of the 1935 Transparent Man’s outer skin is only 0.9 lower than the theoretical maximum value of cellulose triacetate. The outer skin of the Transparent Man from 1962 is even still in almost the same chemical condition as triacetate. The DS for the artificially aged test specimens (Table 6) is discussed below (Section 3.4.4).

#### 3.2.2. Sheet Material

The historical CA sheet material contains the additives dimethyl phthalate (DMP), N-methylbenzenesulfonamide (MBSA), diethyl phthalate (DEP), tris(2-chloroethyl)phosphate (TCEP) and triphenyl phosphate (TPP). The individual additives are very well separated in the total ion chromatogram, so that the peak areas can be integrated (Figure S3). The peak area is a concentration-dependent quantity, which is used in the following as a measure of the additive content. All five additives are commonly used for the preparation of CA. The combination of DMP, DEP and TCEP is quite common [11,56]. DMP, DEP and MBSA serve as plasticizers, while TCEP and TPP, as chlorinated compounds, primarily serve as flame retardants.

### 3.3. Degradation Products

This section explains various observations made on both the naturally aged CA sheet material from the DHMD workshops and the Transparent Figures.

#### 3.3.1. Leaking Liquid

**Figure 12:**
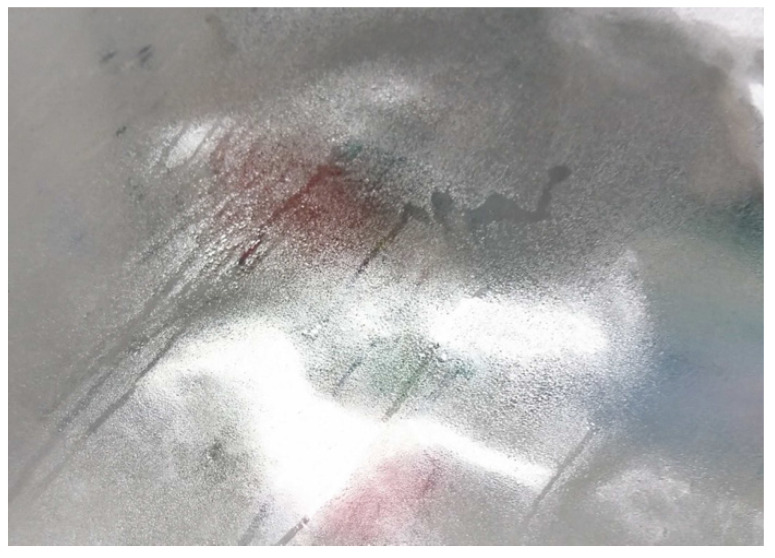
Formation of droplets on CA sheet material from DHMD workshop.

**Figure 13:**
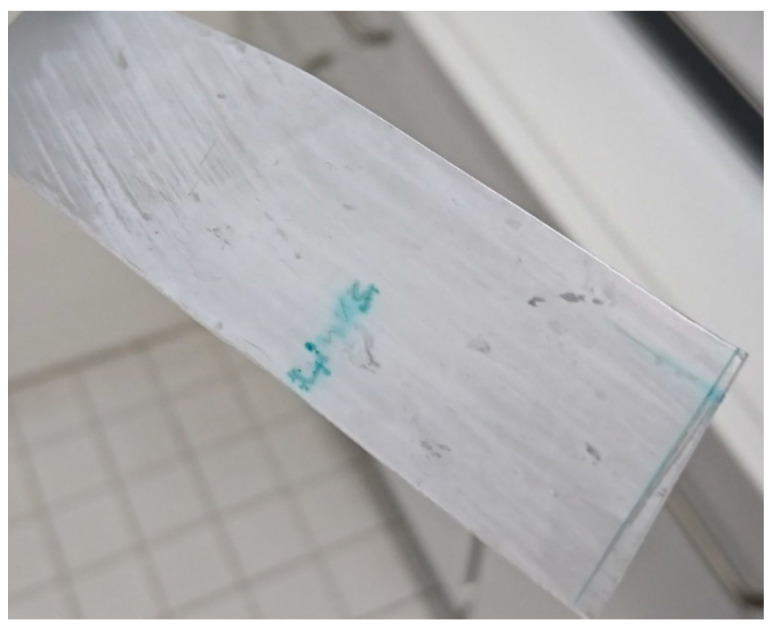
Hazing of CA sheet material at a humidity of below 20% RH.

When storing historic CA sheet material in an actively ventilated hood, alternately dripping and drying occur (Figure 12). Due to the permanent ventilation, a sole influence of acetic acid can be excluded. Rather, it is observed that drop formation occurs at elevated relative humidity. This is the case from values of approx. 50–60% RH at approx. 22–24 °C room temperature. Furthermore, the naturally aged CA sheet material acquires a milky haze when the relative humidity is about 20% and less (Figure 13). The CA becomes transparent again with increasing relative humidity. However, the visual impression of a changed surface remains. The cause of this phenomenon has not been further investigated. No water can be detected in the liquid that emerges from the naturally aged CA sheet material at elevated relative humidity. The pH value of the liquid is lowered, indicating the presence of acid. GC–MS analysis of the liquid confirms the assumption that additives (DMP, DEP, TCEP, MBSA, TPP) are present. DPP as a potential decomposition product of TPP cannot be detected. Accordingly, the escaping liquid is composed most likely of additives and acetic acid.

In the Transparent Cow from 1983, drops form on the CA in the rear right area, which dry off again and reappear depending on the ambient conditions (Figure 6b). It was initially suspected that the drop formation could be related to the concentration of acetic acid, since drop formation or liquefaction of unknown type is also observed in other objects, such as the Transparent Woman from 1935. Comparison of the observations on the Transparent Cow with the climatic values measured in the DHMD shows that drop formation in the object, as with the sheet material, begins at a relative humidity above approx. 50% RH. Only at a later stage, after opening the Transparent Cow, could the already dried crystalline mass be sampled. By means of FTIR, TPP is detected, and thus that it is also a leaked additive.

#### 3.3.2. Acetic Acid in the Air

**Figure 14:**
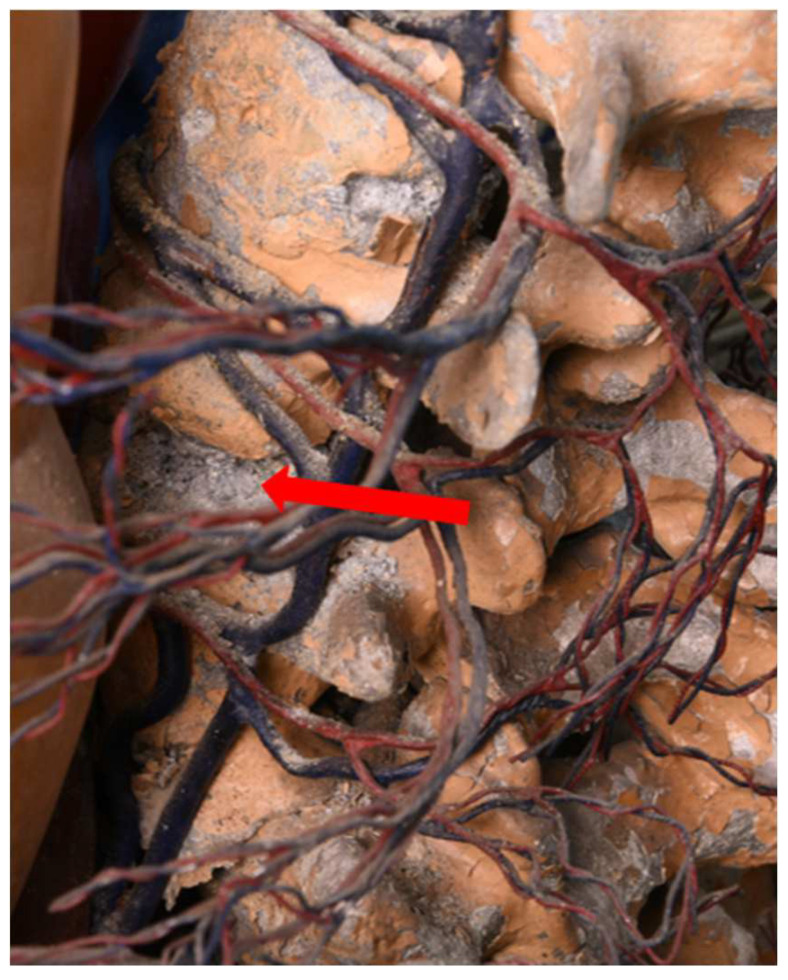
Skeleton elements in the Transparent Man from 1935, made from an aluminum alloy. The arrow points corrosion.

While no acetic acid can be detected on the outside of the Transparent Figures presented above, very different concentrations can be determined on the inside from object to object. Table 7 lists the acetic acid concentrations measured in 2017 and 2018. No acetic acid can be detected in the Transparent Man from 1935. Due to the strong degradation of the outer skin made of CA, it could be that the phase of strong acetic acid emission has already passed or that a quite stable state had been established by the time of the measurements. However, the fact that the figure must have been exposed to high levels of acetic acid at one time since 1935 can be inferred from corrosion on the aluminum skeleton (Figure 14) [45,57]. Furthermore, the outer skin of this object is not completely closed, which is why there is good ventilation and therefore no acetic acid can be detected.

The Transparent Woman from 1935 shows a comparatively high acetic acid concentration of 25 ppm inside. Since this figure and the Transparent Man from 1935 are from the same period and the outer skins are in a similar optical condition, it could be assumed that the acetic acid concentrations are also similar. However, there is one probably crucial difference: the Transparent Woman from 1935, unlike the Transparent Man from 1935, was restored in the early 1990s, when parts of the outer skin were supplemented with new CA [42,46]. The outer skin of this object is completely closed, so that the acetic acid cannot exhaust. It can be assumed that the supplemented CA releases the acetic acid that accumulates inside, while the original CA is in a similar chemical state as the Transparent Man from 1935. The Transparent Man from 1962 has a low acetic acid concentration of 1.3–3 ppm. The exterior of the object is completely closed and is in very good visual condition. Inside, only slight corrosion can be seen on a few ferrous screws. There does not appear to be any aluminum corrosion. This suggests that the chemical condition of the outer skin of CA can also be rated as good.

**Figure 15:**
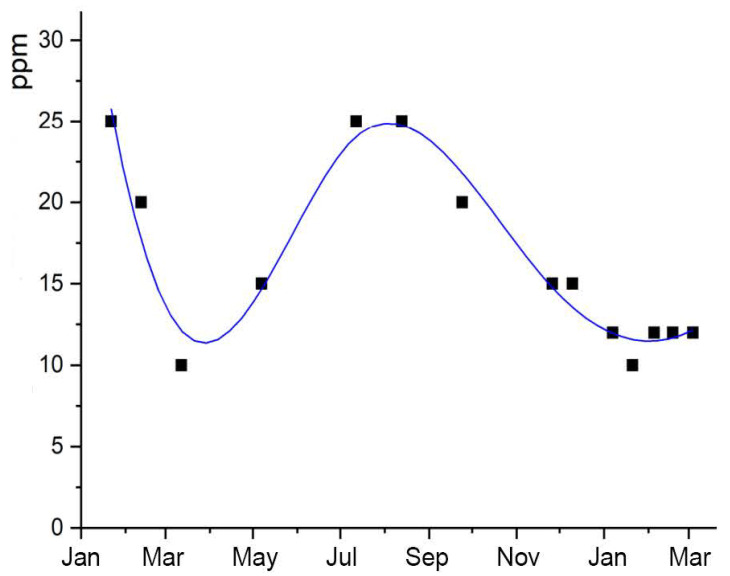
Concentration of acetic acid in the air inside the Transparent Cow of 1983 in the period from January 2018 to March 2019.

**Figure 16:**
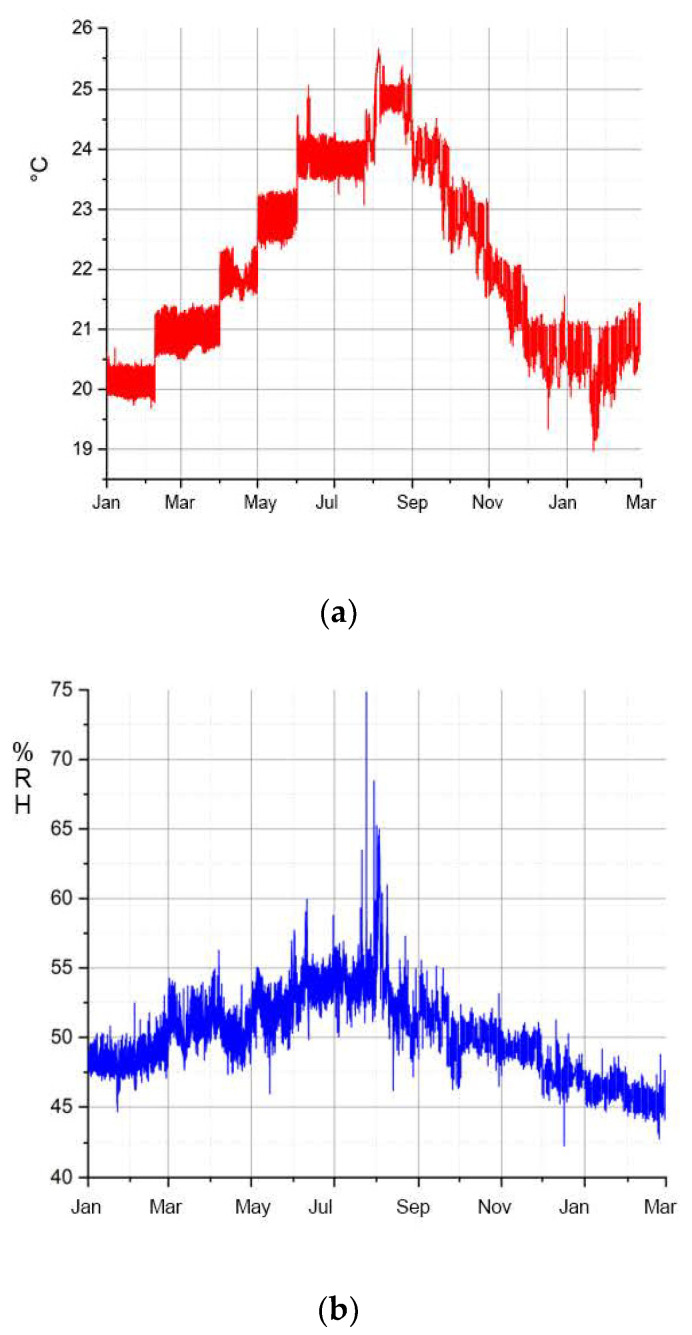
Room climate in the exhibition hall of the Transparent Cow at DHMD in the period from January 2018 to March 2019: (**a**) temperature, (**b**) relative humidity.

In the case of the Transparent Cow from 1983, on the other hand, there are very large fluctuations in the acetic acid concentration inside with 10–25 ppm. Figure 15 shows the annual variation of the acetic acid concentration inside this figure between January 2018 and March 2019. In the winter months, with the exception of January and February 2018, the acetic acid concentration is significantly lower than in the summer months. The exceptional higher values at the beginning of the measurement are caused by previous accumulation, because the inspection flaps were closed before measurement. During measurement, the flaps remained opened, so that the acetic acid concentration and relative humidity adjusted to the exterior since March 2018. In the previous section, a correlation between relative humidity and the migration of additives from the sheet material was described. Figure 16a shows the temperature curve and Figure 16b the RH curve of the exhibition room in which the Transparent Cow from 1983 was located during the period under investigation. It can be assumed that the temperatures inside the cow follow the temperatures measured outside, which are in the range of 20–23 °C in the winter months and 23–25 °C in the months of May–October. The course of the relative humidity lies in the range of 50–55% RH and higher, especially in the summer months. As mentioned above, this is the range of climatic values in which the migration of the additives from the CA sheet material begins. This can also be observed at the Transparent Cow. In contrast, drying of the additives is observed in the drier and cooler winter months (November–January). This suggests a general relationship with relative humidity and temperature. The climatic conditions seem to have a direct influence on the emission of acetic acid, too (see Figure 15), which increases with higher relative humidity and temperature. The assumption that the migration of the additives depends on the acetic acid concentration cannot be confirmed. In fact, both the migration of the additives and the acetic acid concentration depend on the relative humidity and temperature.

### 3.4. Characterization of Artificially Aged CA

#### 3.4.1. Visual Changes

**Figure 17:**
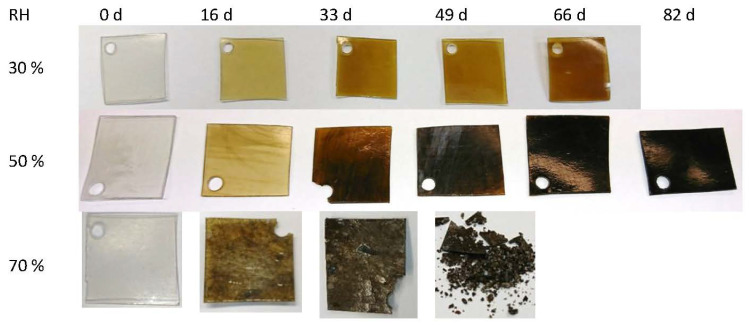
CA test specimens after artificial aging at 70 °C and different humidity levels.

Artificial aging of the CA specimens under variation of the relative humidity leads to visually perceptible changes of the CA (Figure 17). With increasing duration of artificial aging at the same relative humidity, the colorless CA specimens turn increasingly brown to black. At a relative humidity of 70%, cracks and fractures also form and the CA test specimens crumble. At the same duration of artificial aging, the discoloration of the CA test specimens is significantly weaker at lower relative humidity. Already from the optical change of the CA, a slower aging at lower relative humidity can be concluded. Artificial aging of the CA specimens under variation of the acetic acid concentration in the air leads to a clearly different state. The CA specimens remain transparent but show a clearly visible drop formation (Figure 8). An increased acetic acid concentration in the air leads to the escape of a liquid composed of additives and acetic acid. This is also due to the fact that accelerated aging at an elevated temperature is not involved. The relative humidity is kept quite constant at 40–50%, so that the influence of these two factors is greatly reduced.

#### 3.4.2. Color and Brightness

**Figure 18:**
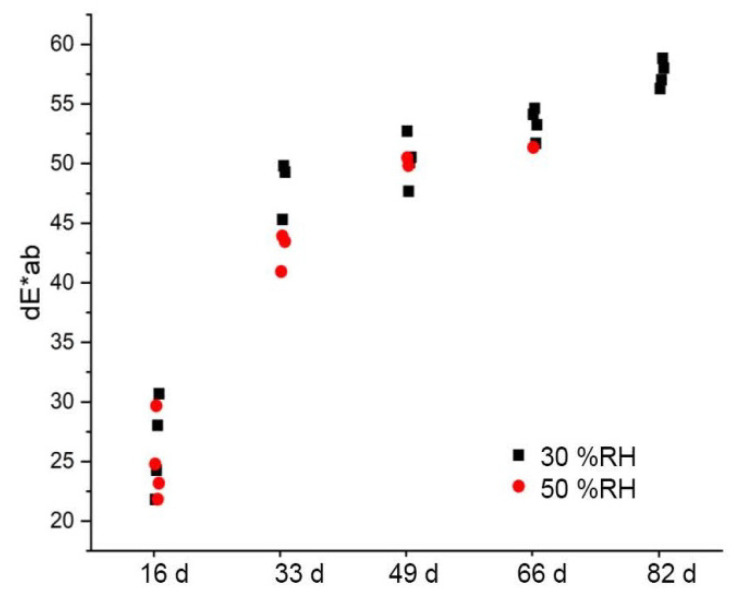
Color difference CIELAB-dE*_ab_ of CA test specimens after artificial aging at 70 °C and different humidity levels compared to non-aged material.

**Figure 19:**
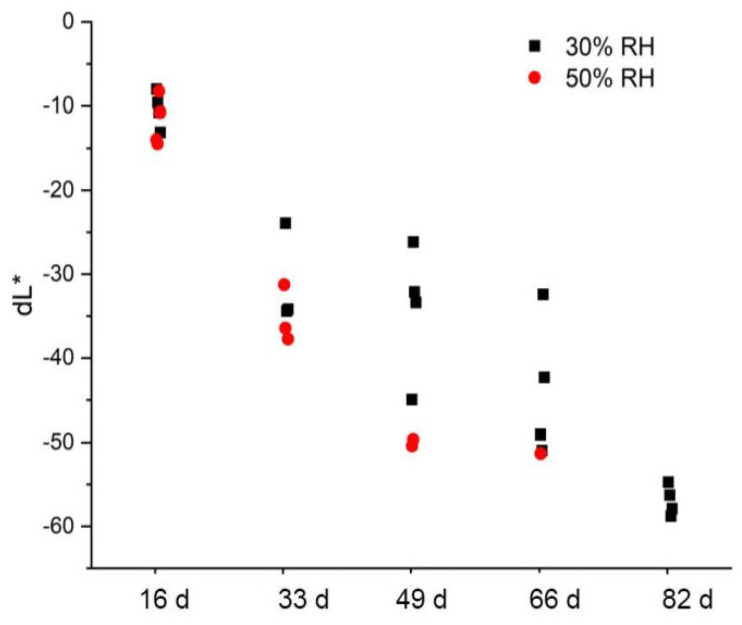
Brightness difference CIELAB dL* of CA test specimens after artificial aging at 70 °C and different humidity levels compared to non-aged material.

The color differences between the artificially aged CA specimens and the non-artificially aged CA specimens are plotted against the aging steps in Figure 18. The color differences compared to the reference are approx. dE*_ab_ = 25–30 after the first aging step and flatten out further on. It cannot be fully explained why the artificially aged CA specimens at a relative humidity of 30% and 50% are calculated to have approximately the same color differences, although they show clear optical differences between them (cf. Figure 17). If, however, only the brightness (dL*) is used as a criterion, the optical difference is confirmed (Figure 19). The brightness decreases with the duration of aging for both variations of relative humidity. As expected, the CA specimens with a clearly darker appearance after artificial aging at 50% RH show a greater loss of brightness.

The decisive factor for the discoloration is the formation of chromophoric groups. For CA, the cause can be both decomposition products of the additives remaining in the material [31] and reactions at the anhydroglucose unit (AGU) itself [58]. The oxidation of the hydroxy groups formed by deacetylation is favored by the elevated temperature of 70 °C and increased relative humidity, so that more chromophoric groups are formed, leading to a different discoloration of the CA specimens as compared to the naturally aged Transparent Figures. Furthermore, due to the very low DS of approx. 0.4 of the CA test specimens, many hydroxyl groups are available at the AGU for oxidation.

#### 3.4.3. Additive Content

**Figure 20:**
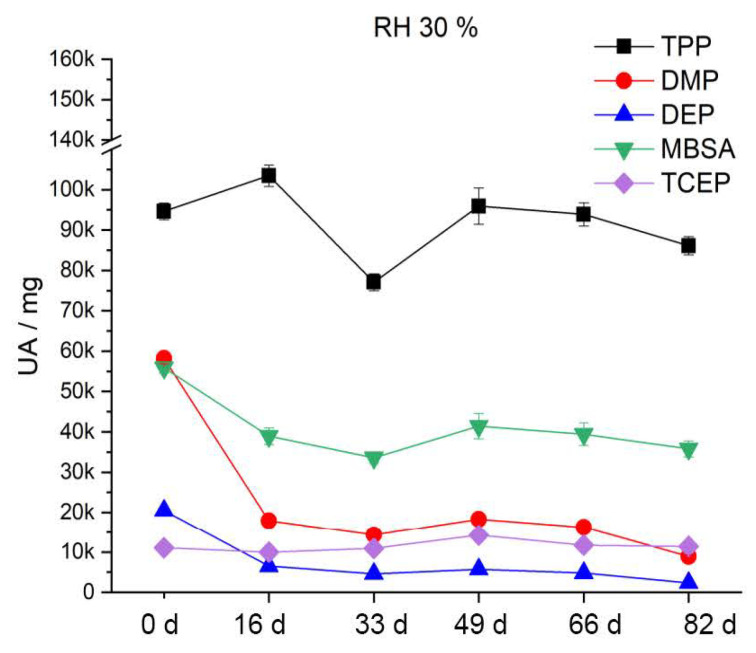
Additive content in the CA test specimens during artificial aging at 70 °C and 30% RH.

**Figure 21:**
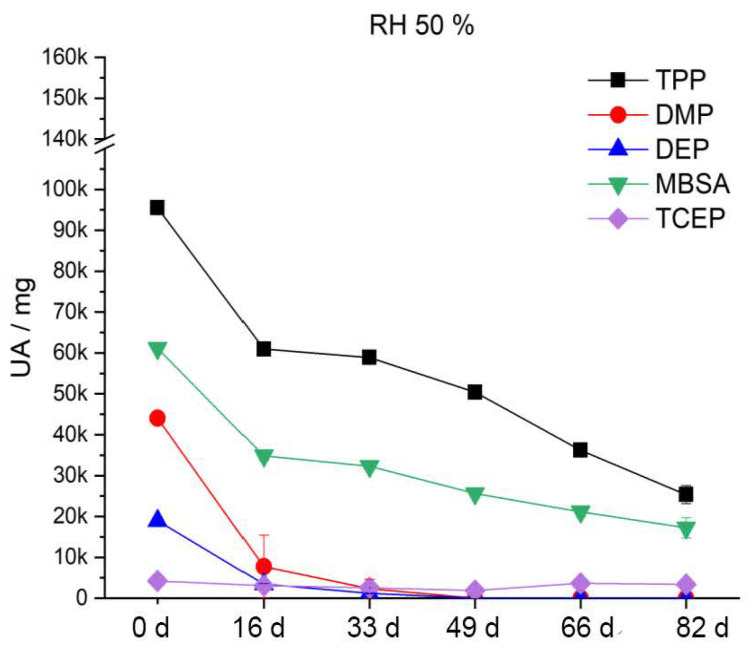
Additive content in the CA test specimens during artificial aging at 70 °C and 50% RH.

**Figure 22:**
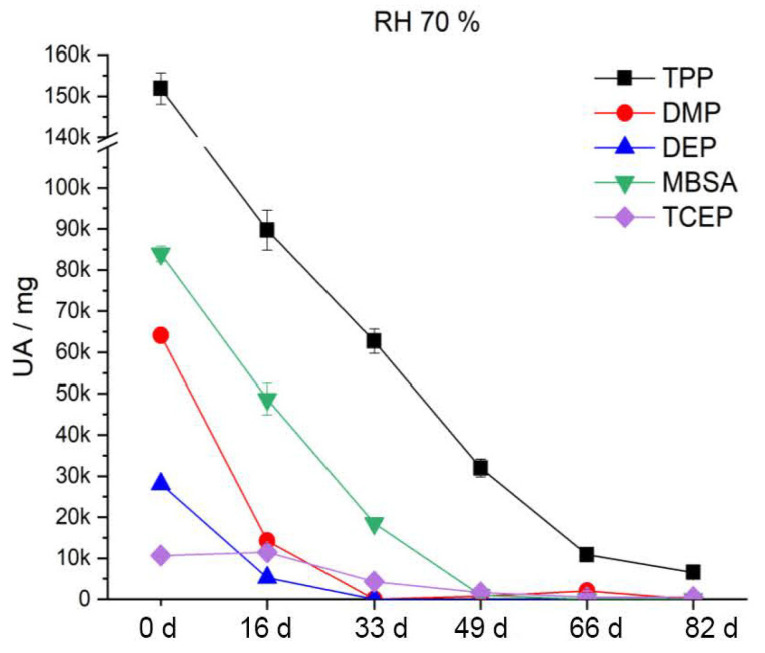
Additive content in the CA test specimens during artificial aging at 70 °C and 70% RH.

After artificial aging at 70 °C and 30% RH (Figure 20), the DMP and DEP content decreases by half after the first aging step. MBSA shows a tendency of a slight decrease with increasing duration of artificial aging. TPP and TCEP, on the other hand, do not significantly leak from the CA under these conditions. Accordingly, phthalates (DMP, DEP) in particular migrate strongly out of the polymer. This finding is supported by the results of Mitchell et al. [59]. After artificial aging at 70 °C and 50% RH (Figure 21), the content of additives is reduced much more strongly and sooner compared to at 30% RH. Already after the third aging step, DMP and DEP are no longer detectable. TPP halves its content by the fifth aging stage. The content of MBSA decreases by two thirds. TCEP shows no significant change. During artificial aging at 70% RH and 70 °C, the additives migrate even more strongly and more readily from the CA. Figure 22 shows that already after the second aging step, DMP and DEP are no longer detectable. MBSA is no longer detectable after the third aging step. After the fifth aging step, about 7% of the original TPP content remains in the polymer. The previously unchanged TCEP decreases to just above the detection limit.

The two phthalates DMP and DEP migrate from the polymer faster and sooner than the other additives, irrespective of the relative humidity. This is attributed in the literature to their high vapor pressure [60]. There is always a sudden decrease from the reference to the first aging stage. In summary, the results show that all included additives migrate more from the CA with an increase in relative humidity. This has an influence on the elasticity of the polymer and explains the severe damage up to crumbling of the CA (Figure 17).

The two additives DMP and TPP are also determined quantitatively. The percentage contents of DMP in the references are approx. 4.5–7% and of TPP approx. 6–11.5%. The decrease in the percentage content during artificial aging under variation of the relative humidity (Figure S4a–c) corresponds to the course already described. As mentioned above, the CA reference specimens are cut from the same contiguous CA sheet, but from different areas. Accordingly, this comes from one manufacturing process and it would have been expected that the percentage contents of DMP and TPP of the total 12 reference samples would be similar. However, specifically for the TPP, this is not the case. One explanation could be that the additives are either already heterogeneously distributed in the material since production or that the CA ages differently. The undefined conditions under which the CA sheet material is stored rather speak for the different degree of aging of the material.

**Figure 23:**
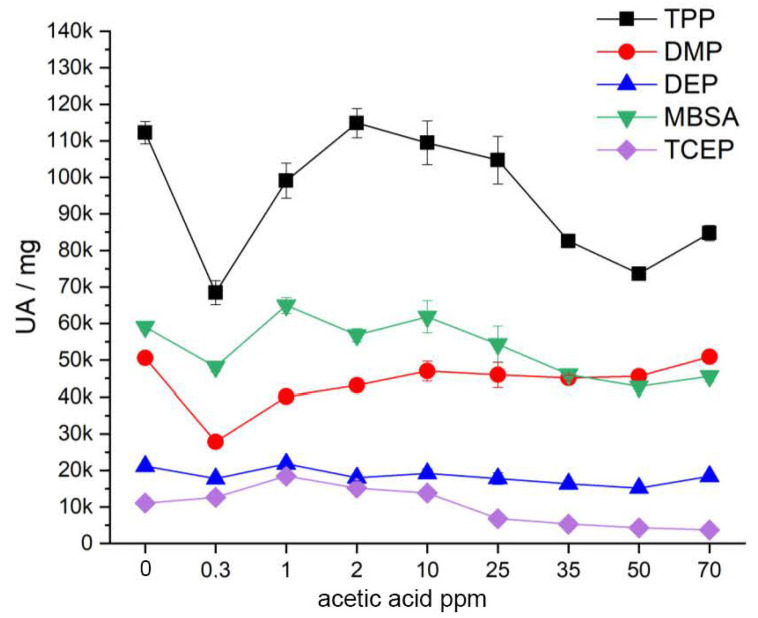
Additive content in the CA test specimens after exposure to air with different content of acetic acid after five weeks.

The development of the additive content after artificial aging of the CA test specimens under variation of the acetic acid concentration is shown in Figure 23. There are no clear trends for DMP, DEP and TPP. The contents of DMP and DEP are stagnant. The content of TPP shows significant fluctuations. MBSA and TCEP show slight tendencies to decrease in content. The results show that an increased acetic acid concentration as the sole influencing factor does not lead to increased migration of the additives. Presumably, this is only caused by the interaction with other factors such as relative humidity and temperature. The strongly decreasing content of TPP, DMP and MBSA at an acetic acid concentration of 0.3 ppm is confirmed once by eight replicate analyses on the same artificially aged CA specimen. Thus, a systematic error can be excluded. It is probably a random inhomogeneity in the already naturally aged CA specimens used for artificial aging. The additives DMP and TPP are further determined quantitatively. The quantitatively determined contents in the references are about 6% for DMP and about 7% for TPP. This corresponds approximately to the contents of the previously determined references for artificial aging (see above).

#### 3.4.4. Degree of Substitution (DS)

**Figure 24:**
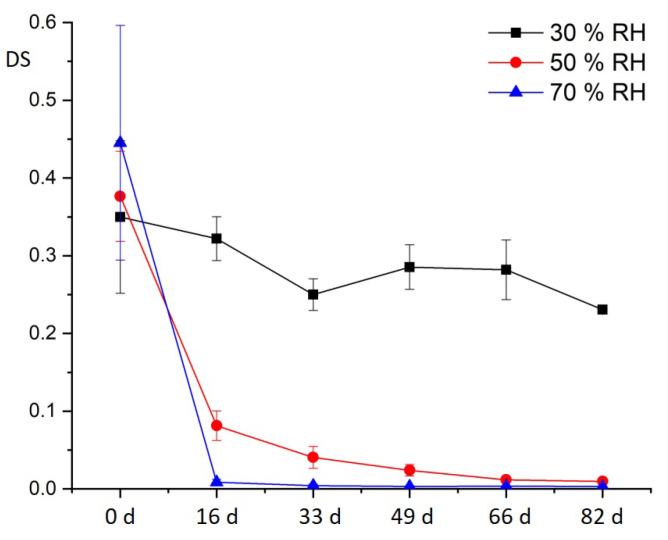
Degree of substitution of CA test specimens during artificial aging at 70 °C and different humidity levels.

Figure 24 shows the DS of CA aged under variation of relative humidity. For CA aged at 30% RH, a tendency of steady decrease in the DS can be seen. There is only a slight difference of 0.1 between the reference and the fifth aging stage (66 days). In contrast, the degree of substitution decreases particularly sharply for CA aged at 50% RH and 70% RH. There, the differences between the reference and the first aging step (16 days) are already 0.3 and 0.4. These decreases correlate with the results of the additive content. The absolute values of the degree of substitution of the naturally aged CA samples (before artificial aging) are very low, about 0.4. To exclude experimental error, the analytical method was validated with two CA products with known DS (see above, 2.5).

The low degree of substitution of the reference is also evident in the analysis of artificially aged CA specimens at different acetic acid concentrations in air. Up to a concentration of 1 ppm, the DS remains the same. Above 2 ppm, the value increases significantly. There could be two reasons for this. The first would be that the acetic acid from the air re-esterifies with the degraded CA. However, no evidence for this can be provided on the basis of the analytical methods applied in this study. Another explanation would be that the acetic acid from the air dissolves in the polymer and is also detected analytically, although the samples were extracted and dried before analysis. However, it cannot be proven that the extraction removes all of the absorbed acetic acid. Nevertheless, the acetyl content, from which the degree of substitution is calculated, apparently increases with the acetic acid concentration in the air. Starting from the reference with an acetyl content of approx. 8%, this increases to approx. apparently 18–20%.

The comparison of the DS of the three investigated figures with the CA specimens shows that the latter are very degraded, with DS = 0.4, although the sheet material was probably produced in the early 1980s and therefore dates from the same period as the Transparent Cow from 1983. The low DS of the sheet material is a surprise, as the optical and mechanical condition did not suggest this. It seems improbable that the one analyzed sheet was a test material originally produced with a low DS, resulting in the determined low value. According to oral communication by remaining employees at DHMD, these sheets would have been used for producing additional figures. The difference is, however, that the sheet material tested here was not used for the production of a Transparent Figure but has since been stored in a cardboard box. Obviously, the very poor ventilation during storage of the sheets in the cardboard box at DHMD (see above, Section 2.1.2) promoted hydrolysis of the acetyl groups. Obviously, open storage with good ventilation is more favorable for the preservation of the acetyl groups. However, due to the undefined storage conditions and the fact that mechanical properties were not the focus of the analysis, no specific statement can be made regarding the correlation of the low DS with the observed condition of the CA sheet material.

#### 3.4.5. Degree of Polymerization (DP)

**Figure 25:**
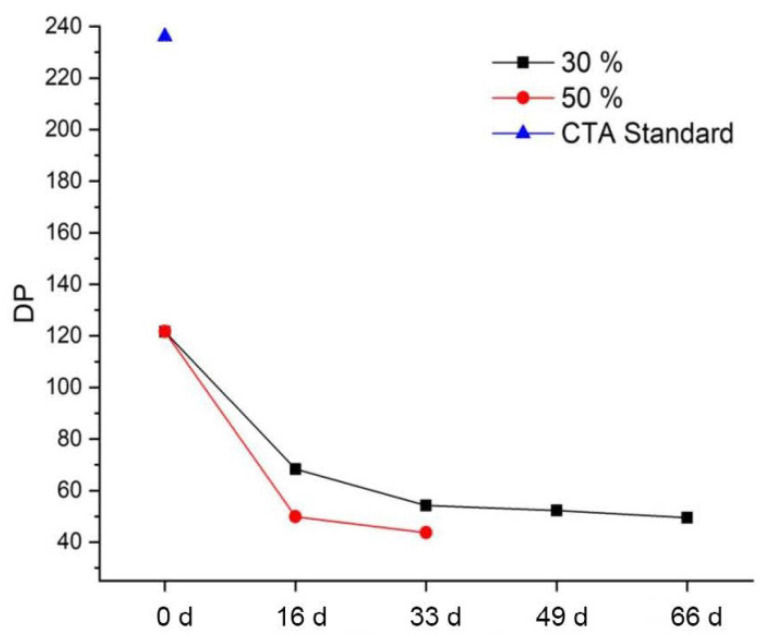
Degree of polymerization of CA test specimens during artificial aging at 70 °C and different humidity levels, and standard sample.

The determination of the DP is carried out exemplarily for selected, artificially aged and already extracted CA samples. Figure 25 shows the results for CA specimens artificially aged under variation of the relative humidity and for the additive-free CTA standard. The DP for the CTA standard is just under 240, whereas the DS of CA lies usually between 150 and 360 [19,61]. The reference of the CA specimens has a DP of about 120. Although the original DP of the reference is not known, it can be assumed that it has already decreased due to natural aging. It decreases very sharply during artificial aging, faster at 50% RH than at 30% RH. The decrease between reference and first aging step (16 days) is stronger than between the following aging steps. The DP for CA specimens artificially aged under variation of acetic acid in air cannot be calculated, since this requires the DS, the determination of which, however, is disturbed, as shown above. Alternatively, the number-average molecular masses can be compared. For the exemplary analyzed CA samples artificially aged under variation of the acetic acid concentration in the air, a slight increase in the number-average molecular mass can be seen (Table 8). Due to the small number of samples, a random distribution cannot be excluded. However, since the values are approximately the same despite the very wide range chosen for the acetic acid concentration in air, it can be assumed that the influence of the acetic acid concentration under the chosen conditions on the DP is only minor.

### 3.5. General Decay and Recommendations for Preservation

The conclusions drawn so far are based on artificial re-aging and the analyses of already naturally aged CA. However, the question arises whether the relative humidity also has such a great influence on newly produced cellulose triacetate or whether it only gains this influence after degradation has already begun. There is much to be said for the latter variant. It is reported that objects made of CA are in an optically stable state for a long time before relatively rapid degradation takes place [23]. The degradation in the Transparent Cow of 1983 proceeded in the same way. This suggests an initiation process that takes some time and is probably not exclusively due to relative humidity. This assumption probably also applies to deacetylation. The catalytic acidic conditions must first develop. An excess of water molecules alone is obviously not sufficient for this. It is therefore conceivable that other external agents, such as temperature, oxygen, light, sulfuric acid as a catalyst residue and other air pollutants, initially cause deacetylation to a very small extent. The acetic acid formed subsequently accelerates ester cleavage as a function of the relative humidity level, resulting in an autocatalytic effect. The measurements of the acetic acid concentration in the Transparent Cow from 1983 confirm the correlation with the relative humidity. Furthermore, progressive degradation (depolymerization, lowering of the degree of substitution) leads to increased moisture uptake [17,62], so that this also promotes deacetylation. Since the relative humidity has both the most serious and the rate-determining influence on the degradation of the CA (additive migration, acetic acid release, depolymerization), both effects are reduced if the relative humidity is kept low. Subsequently, the effects caused by acetic acid release and additive migration (embrittlement, yellowing, shrinkage) are also reduced. The influence of relative humidity on the degradation of historical CA of Transparent Figures correlates with the assumptions of Michalski [39]. This model mathematically describes not only the influence of temperature, but also takes into account relative humidity. However, a simultaneous increase in temperature would cancel out the positive effect of lowering relative humidity. For this reason, both relative humidity and temperature should be lowered in order to slow down the degradation of CA.

For the long-term preservation of the historical CA of the Transparent Figures, a humidity of 30% RH and a temperature of 15 °C are hereby recommended. With Michalski’s model, an almost quadrupling (3.7×) of the “lifetime” is calculated with reference to 20 °C and 50% RH. It should be noted, however, that Michalski’s mathematical model is not unrestrictedly valid and becomes less accurate the further it moves away from 20 °C. However, the humidity should not fall below 30% RH; otherwise, haze will occur on the CA. The recommendation to lower the temperature to 15 °C is based both on practicability in handling the figures and on compatibility for the other materials used, which could not be tested here for temperature sensitivity. However, a significantly lower temperature also has purely practical and technical disadvantages. The display of the Transparent Figures would only be possible in a showcase. A showcase in an exhibition room that is too cool would lead to high energy costs and possibly to undesirable condensation of water. The recommended values for temperature and relative humidity and their primary and secondary effects on degradation are summarized in Table 9.

## 4. Conclusions

The observations and analyses on the Transparent Cow from 1983 and on the naturally aged CA sheet material from the DHMD workshop show that external agents have significant effects on the degradation of CA, among which relative humidity is the most serious. Temperature had no significant effect in these observations. Accordingly, drier environmental conditions lead to a slowing down of the migration of the additives and thus to a longer preservation of the CA.

Discoloration of the CA specimens during artificial aging is to be expected. The CA of the Transparent Figures also discolors in the course of natural aging over the decades. Nevertheless, these have turned more yellowish-green instead of yellowish-brown-black. One reason for this difference may be the elevated temperature of 70 °C over several weeks during artificial aging. Therefore, the reactions in the CA specimens are not only faster, but presumably also enhanced.

Both under real museum conditions and under laboratory conditions with good ventilation, a visible leakage of additives from the examined CA materials occurs at a relative humidity above 50–60%. Below 50% RH, the additives dry out. Temperature had no significant effect on these observations. This observation is supported by GC–MS analyses on naturally-aged CA sheet materials artificially re-aged under an elevated temperature of 70 °C with variation of relative humidity. The five additives contained (DMP, DEP, TCEP, MBSA, TPP) migrate significantly faster from the CA at elevated relative humidity. The migration rate decreases with 70% RH > 50% RH > 30% RH. The formation of hydroxy groups as a result of deacetylation changes the intermolecular interactions between the additives and the polymer chains. Water can be preferentially incorporated, and the additives migrate out of the polymer to a greater extent. However, the interactions between the additives and the polymer chains can also change due to subsequent reactions of the hydroxyl groups such as oxidation to ketones, aldehydes and carboxylates [63].

The effect of relative humidity on the CA sheet material has been shown to reduce the degree of substitution (DS) of the CA specimens artificially aged under variation of relative humidity [24,25]. This produces acetic acid, which accumulates in the air and can be measured in the Transparent Figures and has further effects. The comparison of the sheet material made of CA from the DHMD workshop with the original CA of the Transparent Figures shows that, with regard to the additive composition, it makes sense to use the plate material made from CA for artificial aging, since very close-to-application results are obtained. Even the CA sheet material with a very low DS of 0.4 gives very clear results in artificial aging with respect to the influence of relative humidity.

In addition to additive migration, deacetylation and discoloration, the relative humidity also has an influence on the degree of polymerization (DP) of the CA test specimens. This decreases faster with increasing relative humidity. The sole influence of acetic acid on the degradation of the CA specimens appears to be small. In contrast to artificial aging under variation of relative humidity, no increased additive migration with increasing acetic acid concentration in the air can be observed. Likewise, no significant decrease in the degree of polymerization can be proven. This is in contrast to descriptions in the literature [3,18]. The reason could be that the experimental setup limits the concentration of water molecules in the desiccators as reaction partners for hydrolysis.

For the long-term preservation of the historical Transparent Figures, values for relative humidity (30% RH) and temperature 15 °C are recommended (Table 9). An overall concept for preservation and, furthermore, restoration of the Transparent Figures is published in a collective volume [64].

## Figures and Tables

**Table 1:** Sampling areas and mass of samples taken from the original Transparent Figures.

Object	Sampling Point	Mass
Transparent Man from 1935	Weld right side of upper part of the body	4.5 mg
Transparent Man from 1962	Gap on the neck behind left inspection flap	5.0 mg
Transparent Cow from 1983	Gap on the neck right side behind inspection flap	6.6 mg

**Table 2:** Overview of the material used for analysis.

	FTIR-/Raman Spectroscopy	Development of Ultrasonic Extraction [51]	Artificial Aging	Color Measurement	Additive Content	DS	DP
CA sheet material	X	X					
CAB sheet material	X						
CA test specimens gained from sheet material			X	X	X	X	X
original CA sampled from selected Transparent Figures	X				X	X	
CTA standard						X	X
cigarette filter (standard)						X	

**Table 3:** Degree of substitution (DS) determined for additive-free CA reference material.

Material	DS (Lit.)	Experimental DS (Mean)	Standard Deviation of DS
CTA standard	3	3.02	0.21 (7.0%)
Cigarette filter	c. 3	2.90	0.22 (7.6%)

**Table 4:** Relative reaction rates of aging of CA related to 20 °C and 50% RH, calculated after Michalski [39].

RH	Relative Reaction Rate @20 °C	Relative Reaction Rate @70 °C
30%	0.51	114
50%	1.00	222
70%	1.55	344

**Table 5:** Theoretical aging time for CA under artificial aging at 70 °C and different humidity levels, as calculated after Michalski [39] using the relative reaction rates as in Table 4.

Aging Time at 70 °C	16 d	33 d	49 d	66 d	82 d
RH	Calculated Aging Time at 20 °C (Years)				
30%	5.1	10.2	15.3	20.4	25.5
50%	10.0	20.0	30.0	40.0	50.0
70%	15.4	30.8	46.2	61.6	77.0

**Table 6:** Degree of substitution of CA from the outer skin of selected Transparent Figures and CA test specimens.

Material	DS
Transparent Man from 1935	2.1
Transparent Man from 1962	2.9
Transparent Cow from 1983	2.7
CA test specimens	0.4

**Table 7:** Concentration of acetic acid in the air inside the Transparent figures in the years 2017 and 2018.

Figure	Acetic Acid (ppm)	Number of Measurements
Transparent Man from 1935	0	3
Transparent Woman from 1935	25	2
Transparent Man from 1962	1.3–3	3
Transparent Cow from 1983	10–25	20

**Table 8:** Number average molecular masses (Mn) of CA test specimens aged under different levels of acetic acid in air for five weeks.

Acetic Acid in Air [ppm]	Mn [g/mol]
2	30,000
35	31,500
70	33,000
CTA Standard	68,000

**Table 9:** General recommendations for climate conditions for storage of Transparent Figures and their consequences.

Recommendation	Primary Consequences	Secondary Consequences
temperature:	- increase in lifetime approx. 4× (compared to 20 °C/50% RH) [39] - avoiding leakage of plasticizer - considerable reduction of acetic acid emission - considerable reduction of depolymerization	Less damage to other constituents retardation of: - shrinkage - embrittlement - yellowing
optimal: 15 °C		
maximum: 20 °C		
RH:		
optimal 30%		
maximal: 50%		

## Data Availability

The data that support the findings of this study are partially published in [1] and available on request from the corresponding author.

## Supplementary Materials

The following supporting information can be downloaded at: https://www.mdpi.com/article/10.3390/polym15132838/s1, Figure S1: Calibration curve for GC–MS analysis of the additives DMP and TPP; Figure S2: Calibration curve for GC–MS analysis of the degree of substitution; Figure S3: Total ion chromatogram (TIC) of the methanol extract of naturally aged CA sheet material; Figure S4: Quantitative additive content in the CA test samples during artificial aging at 70 °C and different humidity levels.
