# Supplementary material for: Transparent Figures: Researching and Preserving Objects of Cellulose Acetate
Source: Polymers (Basel). 2023 Jun 27;15(13):2838. doi: 10.3390/polym15132838 (PMC10346856; doi:10.3390/polym15132838)
Supplement: Supplementary file 1 [file polymers-15-02838-s001.zip › polymers-2394951-supplementary.pdf]

Transparent Figures: Researching and Preserving Objects of Cellulose Acetate
Benjamin Kemper, Christoph Herm

Supplementary material

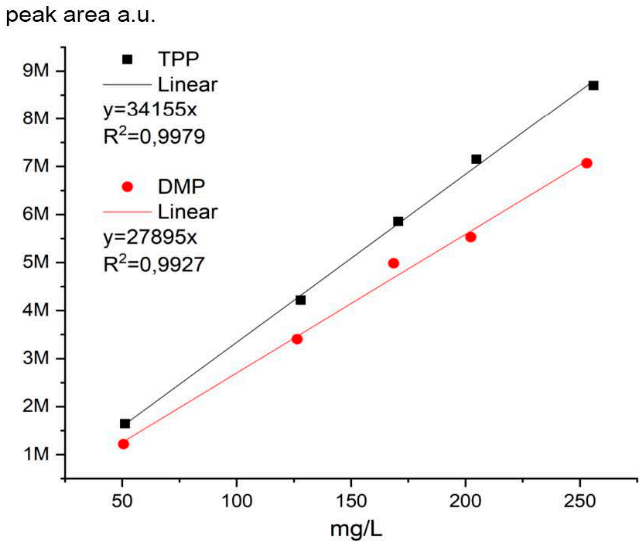

Figure S1. Calibration curve for GC-MS analysis of the additives DMP and TPP.

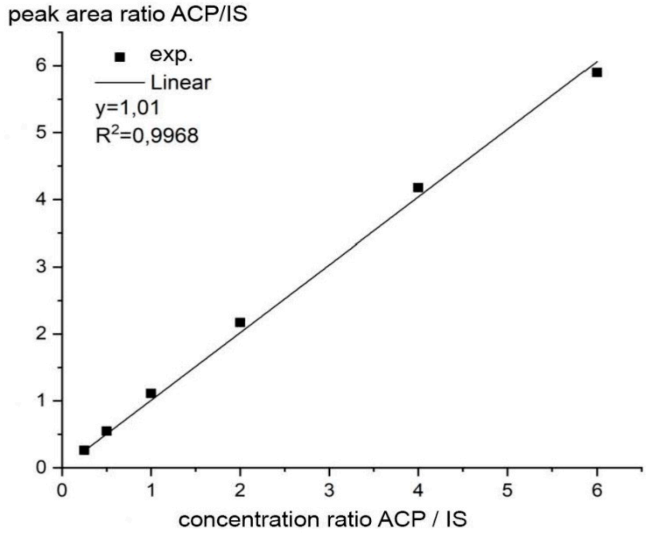

Figure S2. Calibration curve for GC-MS analysis of the degree of substitution. ACP = acetyl pyrrolidine, IS = internal standard (N-methyl-2-pyrrolidone)

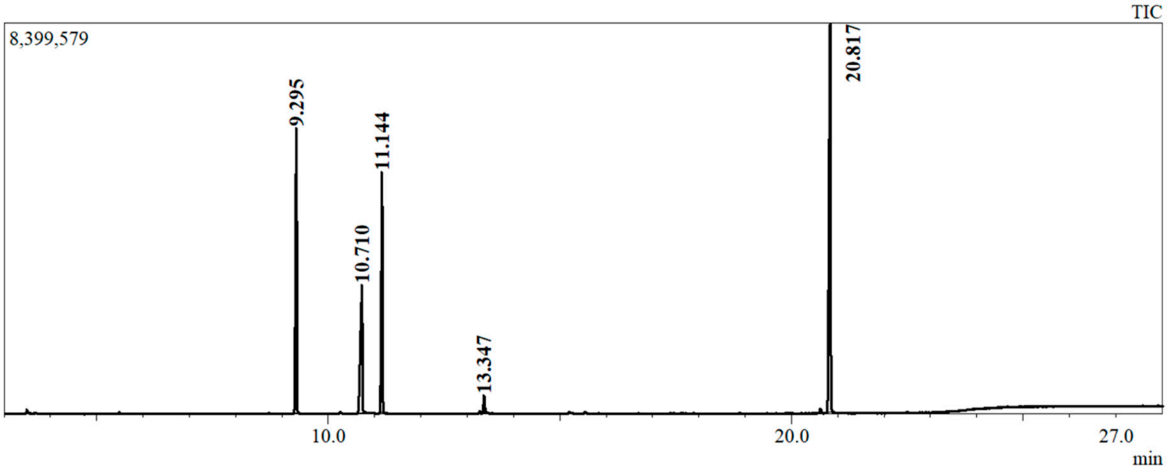

Figure S3. Total ion chromatogram (TIC) of the methanol extract of naturally aged CA sheet material.

Additives: DMP (9.295 min), MBSA (10.710 min), DEP (11.144 min), TCEP (13.347 min), TPP (20.817 min)

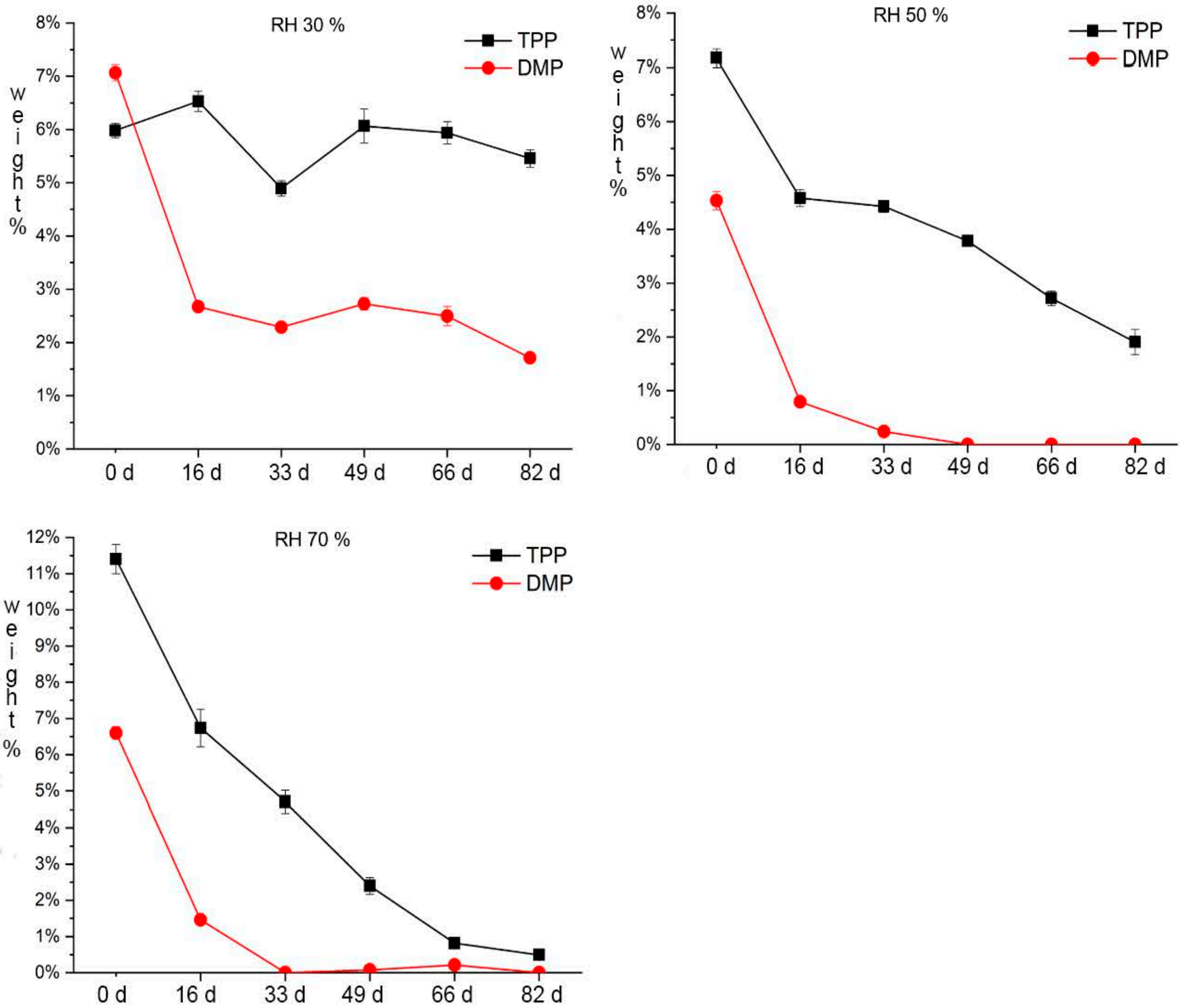

Figure S4. Quantitative additive content in the CA test samples during artificial aging at 70 °C and different humidity levels: a) 30 %RH b) 50 %RH c) 70 %RH
